# Mental Practice Combined with Physical Practice to Enhance Hand Recovery in Stroke Patients

**DOI:** 10.1155/2014/876416

**Published:** 2014-11-09

**Authors:** Hua Liu, Lu-ping Song, Tong Zhang

**Affiliations:** ^1^Capital Medical University School of Rehabilitation Medicine, China Rehabilitation Research Center, No. 10 Jiaomenbei Road, Beijing 100068, China; ^2^Capital University of Physical Education and Sports, School of Kinesiology and Health, Beijing 100191, China

## Abstract

*Objectives.* To evaluate whether combining mental practice with physical practice training enhances hand function in patients with stroke. *Methods.* 10 for treatment and 10 for control were recruited for this pre/posttraining matched case control study. In the treatment group, subjects underwent combining mental practice with physical practice for four weeks. In the control group, subjects only participated in physical practice. Change of hand function and the number of activated voxels of the contralateral somatosensory motor cortex (SMC) acquired by functional magnetic resonance imaging were measured. *Results.* After training, the Action Research Arm Test score increased by 12.65 for treatment and by 5.20 for control. There was a significant difference in the Action Research Arm Test score between the two groups (*P* = 0.04). The activated voxels number of the contralateral SMC increased in both groups, but the activated voxels number in the contralateral SMC and the improvement of hand function for treatment were greater than for control. In the treatment group, the number of activated voxels of the contralateral SMC was positively correlated with better hand function scores. *Conclusions.* Combining mental practice with physical practice may be a more effective treatment strategy than physical training alone for hand recovery in stroke patients.

## 1. Introduction

Up to 85% of stroke survivors experience hemiparesis, resulting in impaired movement of the arm and hand [1]. Among these survivors, a large proportion (46% to 95%) remains symptomatic six months after the ischemic stroke event [2]. Loss of arm function adversely affects quality of life [3], and functional motor recovery in affected upper extremities in patients with hemiplegia is the primary goal of physical therapists [4]. Continuous rehabilitation training following subcortical damage in movement disorders can achieve motor function recovery [5]. However, due to the impairment of movement function, the patient's capacity for independent movement is partly and sometimes completely lost and active training therapies are thus limited. Intensive rehabilitation is expensive, and many rehabilitation centers provide clients with a limited number of therapy sessions before discontinuation of rehabilitation financing. Given these limitations, we are committed to developing strategies that will minimize the use of costly resources and maximize practice opportunities to enable functional motor learning and recovery [4].

Motor imagery (MI) is a mental process of rehearsal for a given action in order to improve motor function [6]. And mental practice (MP) is a training method during which a person cognitively rehearses a physical skill using MI in the absence of overt, physical movements for the purpose of enhancing motor skill performance [7]. Recently, research has shown that MP using MI (MP_MI) combined with physical practice (PP) can promote recovery of motor function [8–10]. The therapeutic benefit of MP_MI was demonstrated for dyskinesia rehabilitation [11] and gait training in chronic stroke patients [12]. In acute stroke patients, Page et al. [10] showed that the Fugl-Meyer assessment (FMA) score and the Action Research Arm Test (ARAT) score did not significantly improve after six weeks with PP alone. However, combining MP_MI and PP increased FMA and ARAT scores by 13.8 and 16.4, respectively. In patients with chronic stroke, MP_MI combined with occupational therapy improved FMA score in the upper extremities greater than occupational therapy alone [10]. Indeed, MP_MI as a special motor skill activated the same muscles and neural areas as PP [10].

With technological advances in functional magnetic resonance imaging (fMRI), interest regarding MI began to grow. Previously, it was shown that MI and motor execution (ME) activated similar areas of the brain, such as the premotor cortex [13] and the supplementary motor area (SMA) [14]. Stinear et al. [15] applied transcranial magnetic stimulation over contralateral primary motor cortex (M1) to elicit motor evoked potentials in the dominant abductor pollicis brevis during kinesthetic MI and further gave other line of evidence on MI and ME involving overlapping neural structures. However, MI and ME shared some different cortical networks. Sharma and Baron [16] considered that MI and ME both shared the contralateral M1, the premotor cortex, parietal areas, and SMA. ME exclusively involved the contralateral M1, the primary somatosensory cortex (S1), and the ipsilateral cerebellum whereas MI exclusively involved the ipsilateral M1 and the premotor cortex. A meta-analysis revealed that MI consistently recruited a large frontoparietal network in addition to subcortical and cerebellar regions [17]. The involvement of M1 during MI was less consistent [18]. Some studies reported a lack of activation of M1 during MI in contrast to ME in healthy participants [19]. Other studies had detected a slight increase of activity in M1 during MI, albeit with a lower intensity than that during ME [20]. Solodkin et al. [21] suggested that M1 was active during kinaesthetic imagery, but not during visual imagery. Gerardin et al. [22] showed individual heterogeneity in the activation of M1 during MI that may be related to individual differences in the activity of MI.

Given the evidence regarding motor function and cortical reorganization with MP_MI application, MP_MI might be a noninvasive and effective rehabilitation strategy for patients with stroke [23]. A fMRI study on a ME task of finger-thumb opposition showed that compared with the unaffected hand, the ratio of contralateral to ipsilateral sensorimotor cortex (SMC, M1, and S1) activity during ME of the affected hand increased significantly over time as the affected hand regained function [24]. Affected-hand MI activation was to affected-hand ME. Unlike control healthy subjects and the unaffected hand, activation in M1 (BA4a) and dorsal premotor was not lower during affected-hand MI as compared with affected-hand ME [25]. It is our hypothesis that MP_MI combined with PP would enhance hand function in patients with stroke relative to PP alone and that using MI of the affected hand would activate more areas of motor cortex than using MI of the unaffected hand. In this case-control study, we detected hand function changes in patients with stroke after four weeks of training and tested with fMRI the number of activated voxels in contralateral SMC in the following conditions: ME using the affected hand, MI using the affected hand, ME using the unaffected hand, and MI using the unaffected hand. We explored the effect of MP_MI on the recovery of hand function and characterized the pattern of motor imagery best for the recovery of hand function in stroke patients undergoing MP_MI training.

## 2. Methods

### 2.1. Subjects

This study was approved by the China Rehabilitation Research Center Ethics Committee, and the study had been registered in Chinese Clinical Trial Registry (registration number ChiCTR-OCH-12002238). In total, 20 patients were enrolled, and written informed consent was obtained in accordance with the Declaration of Helsinki. Inclusion criteria included patients with a subcortical, first-ever stroke with neurological deficit in the affected upper limb (nadir hand function level beyond Brunnstrom III) and no significant cognitive impairment. Exclusion criteria included [25] carotid artery stenosis/occlusion, persistent language deficit, neglect/inattention, significant renal/liver disease, treatment with selective serotonin reuptake inhibitors/benzodiazepines, visual impairment, depression, left-handedness, and contraindications to MRI.

All subjects were right-handed, as assessed by the Edinburgh scale [26]. 20 patients were randomly allocated to the treatment and control group. Ten subjects (5 female; mean age, 48.90 ± 7.19 years; average duration from stroke, 1.91 ± 0.80 months; average score for action research arm test (ARAT) before training, 17.50 ± 0.83) were assigned to the treatment group and underwent MP_MI plus PP training. The other ten subjects (4 female; mean age, 53.10 ± 10.38 years; average duration from stroke, 1.83 ± 0.64 months; average score for ARAT before training, 15.10 ± 15.59) were enrolled in the control group and underwent PP training alone. The rating scale of the kinesthetic and visual imagery questionnaire (KVIQ) [27] assessed the vividness of each dimension of MI on a five-point ordinal scale and was adapted in healthy adults and for persons with physical disabilities. This questionnaire had been translated into Chinese by the consent of Malouin. The Chaotic Motor Imagery Assessment (CMIA) was defined as an inability to perform MI accurately or, if having preserved accuracy, the demonstration of temporal uncoupling [28]. Including such subjects with CMIA in fMRI studies would produce incongruent results [25]. All subjects passed the assessment of KVIQ (KVIQ ≥ 25 score) [27] and CMIA [29]. The clinical and demographic data for all subjects is presented in Table 1.

### 2.2. Experimental Procedure

Subjects underwent the remainder of the measurement battery (baseline measure point) before beginning the intervention on that or the following day. Intervention was given once each working day for four weeks (20 days). Subjects performed the measurement battery, except for the CMIA, on the day of or the day after the last treatment (outcome measure point). ARAT score [30] was used to assess hand function. The physiotherapists who assessed the ARAT score were blinded to all subject group assignments.

### 2.3. Intervention

The treatment group and the control group received PP training (mainly using the NeuroDevelopmental Treatment-Bobath (NDT-Bobath) method, as described previously [31]). The PP training was implemented once a day for 45 minutes from Monday to Friday for four consecutive weeks.

The treatment group received MP_MI training in addition to PP training. Implementation of MP_MI training was as described in Simmons et al. and obtained by their consent [29]. The MP_MI training was implemented once a day for 45 minutes from Monday to Friday for four weeks. Each training consistent of three sessions with five minute breaks between two sessions. Each MP_MI session was performed by an appropriate position, followed by explanation of rules and instructions by the physiotherapist. The rules of the MI task were explained as imagining in the first person. During performing the MI task, the subjects imagined themselves performing an instructed movement without actual movement. The subject received the following instructions before each session: during this session there are some MI activities including flexion/extension of the thumb, abduction/adduction of all digits, making a fist/spreading the hand, moving extended fingers backwards and forwards, and moving the hand between the ulnar and radial deviation that you are going to imagine doing with your paretic hand. Each MI activity was performed as follows: firstly, the physiotherapist explained the MI activity and watched the video of the MI activity twice with the subject. Secondly, the subject used the unaffected hand to physically perform the MI activity twice. Thirdly, the subject imagined the MI activity using the unaffected hand. The instructions of each MI activity were given as “Close your eyes. Concentrate on your hand, but do not move it. Concentrate on how it feels just resting there. Do not move your fingers, hand, or arm. Just imagine the required MI activity (e.g., making a fist, you can imagine relaxing your five fingers of the unaffected hand, letting the palm toward the desktop, slowly and slowly flexing your five fingers…slowly…till making a fist…feeling a power fist and then slowly relaxing your five fingers…till extending your fingers and letting the palm toward the desktop) and do not move anything. Open your eyes when you have done this action two times.” Lastly, the subject imagined the MI activity using the affected hand three times. The same verbal instructions were given for the affected and unaffected hand.

The control group program was matched for the duration of the sessions with the treatment group program. The first two steps in the control group were the same as in the treatment group. The third step of the MI activity with the unaffected hand was the relaxation activity, and the last step of the MI activity with the affected hand was also the relaxation activity [9]. The total duration in the control group was the same as in the treatment group.

### 2.4. fMRI Experimental Procedure

For fMRI part of the study, we employed a block design with an auditory-paced (1 Hz) thumb-palm opposition sequence. The thumb-palm opposition task was performed as follows: thumb started from the rest position, touched the palm in an opposition sequence at a speed of one time per second, and then came back to the rest position. All subjects performed the task of ME or MI using the affected/unaffected hand inside a magnetic resonance (MR) scanner during a pretraining session that followed a brief familiarization period. During pretraining, four scanning sessions were completed: ME using the affected hand firstly, MI using the affected hand secondly, ME using the unaffected hand thirdly, and MI using the unaffected hand lastly. The sessions of ME using the affected/unaffected hand were performed by the subjects physically performing the movement of thumb-palm opposition. The sessions of MI using the affected/unaffected hand were performed by the subjects mentally imagining the movement of thumb-palm opposition. On the day following the four weeks of training, the subjects returned to the MR scanner for a posttraining session, using the same conditions as pretraining. Brain activation was measured with blood oxygen level dependent (BOLD) fMRI. Each session was 6.24 min long, including run-up for 24 s, ME or MI of thumb-palm opposition for 30 s, and rest for 30 s. The thumb-palm opposition task and rest was considered one cycle, with a total of six cycles per session.

For MI paradigm, subjects were instructed to mentally rehearse thumb-to-palm opposition movements by a prerecorded voice that said “imagery,” and to change to the rest condition when the voice said “rest.” Auditory prompts were presented through sound-insulated earphones connected to the computer's audio output.

For ME paradigm, subjects were instructed to perform the requested movement by a prerecorded voice that said “motion,” and to change to the rest condition when the voice said “rest.” During fMRI scanning, room lights were dimmed and subject's eyes were closed.

#### 2.4.1. Data Acquisition

A 1.5-T GE Signa system (Chalfont St. Giles, UK) was used to acquire both T1-weighted imaging (T1WI) anatomic images and BOLD-fMRI. The parameters used were as follows: Fourier-acquired steady-state technique sequence (FAST) (TR 200 ms), TE 24 ms, FOV 240 mm × 240 mm, matrix 320 × 192, and slice thickness 5 mm. The pulse sequence for fMRI scans was a T2^*^-weighted echo-planar imaging (EPI) sequence, with a TR/TE 3000 ms/40 ms, 90° flip angle, and matrix 64 × 64. The parameters for FOV and thickness were the same as the anatomical imaging.

#### 2.4.2. Image Processing

fMRI data analyses were performed by trained technicians blind to subject identity and group membership. Image preprocessing and statistical analyses were performed using SPM8 (Wellcome Department of Cognitive Neurology, Queen's College, London, England) and MATLAB (Mathworks, Inc., Natick, MA, USA). Preprocessing included realignment, normalization, and smoothing. In any given subject, head movement was no more than 2 mm. The unilateral *t*-test analysis of SPM8 was used, respectively, under the four conditions: affected hand execution, affected hand imagination, unaffected hand execution, and unaffected hand imagination. For generation of the activated image, the threshold for voxel detection was set at 10 voxels.

### 2.5. Statistical Methods

Differences in hand function and the number of activated contralateral SMC voxels before and after training were determined using a paired *t*-test. Analysis of variance (ANOVA) was performed to compare a main effect for 2 groups (treatment group and control group) × 4 conditions (ME using affected hand, MI using affected hand, ME using unaffected hand, and MI using unaffected hand) for the number of activated voxels in the SMC. Post-hoc was performed to compare the effect between two conditions. After training, the Spearman rank correlation analysis was performed between the number of activated contralateral SMC voxels and the change in hand function score. *P* < 0.05 was considered statistically significant. Correction for multiple comparisons was performed using a false discovery rate (FDR) of 0.05. All statistical analyses were performed using SPSS 17.0 (SPSS Inc., Chicago, IL, USA).

## 3. Results

### 3.1. General Data Prior to Training

There was no statistically significant difference between the two groups in age (*P* = 0.307, df = 18), the time since stroke (*P* = 0.808, df = 18), and ARAT score (*P* = 0.538, df = 18) prior to the onset training.

### 3.2. Hand Function Changes between Two Groups after the Training

Hand function score, as measured with the ARAT, was significantly higher after training than before the training (*P* < 0.01, df = 18, Table 2) in both groups. However, this improvement was significantly greater in the combined treatment group over control (*P* < 0.05, df = 18, Table 2).

### 3.3. Activation Areas of ME and MI Using Affected and Unaffected Hand in Both Groups before and after Training

**Figure 1:**
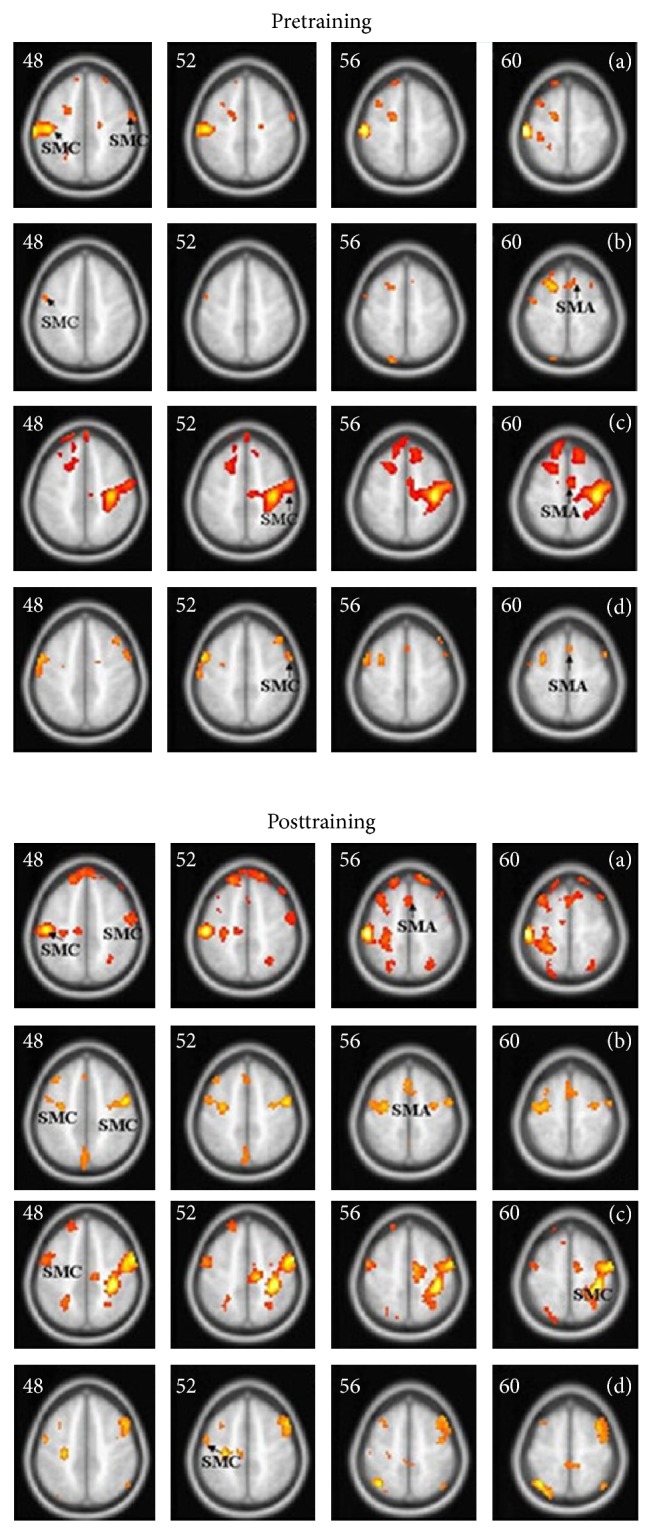
The activated areas of pretraining and posttraining using affected hand execution (a), affected hand imagination (b), unaffected hand execution (c), and unaffected hand imagination (d).

Before training, ME using the affected hand mainly activated bilaterally SMC, SMA, and cerebellum. MI using the affected hand mainly activated the bilateral SMA, the contralateral SMC, and the bilateral cerebellum. ME using the unaffected hand mainly activated contralateral SMC and ipsilateral SMA. The activated area for MI using the unaffected hand was much smaller than the first three conditions and was mainly bilateral SMA and ipsilateral SMC (Figure 1).

After training, ME using the affected hand mainly activated the contralateral SMC and the bilateral SMA. MI using the affected hand mainly activated the contralateral SMA, the contralateral SMC, and the ipsilateral SMA. ME using the unaffected hand mainly activated the contralateral SMA, the contralateral SMC, and the ipsilateral SMC. MI using the unaffected hand mainly activated the bilateral SMA and the ipsilateral SMC (Figure 1).

### 3.4. Comparison of the Number of Activated Voxels in Contralateral SMC in the Four Conditions between Both Groups before and after Training

Before training, there was no significant difference between the two groups in the number of activated voxels in contralateral SMC (*F*_group(1,75)_ = 0.010,*  P* = 0.922, Table 2). There was a significant main effect for the number of activated voxels among the four conditions (ME using unaffected hand, ME using affected hand, MI using affected hand, and MI using unaffected hand) (*F*_condition(3,75)_ = 65.635, *P* < 0.01, Table 2). Post-hoc comparisons revealed that the number of activated voxels was significantly greater for ME using affected hand execution than for MI using the affected hand (*P* < 0.05). The number of activated voxels was significantly greater for MI using affected hand imagination than for MI using unaffected hand (*P* < 0.05). The number of activated voxels in the contralateral SMC in the four conditions is shown in Table 2.

After training, the number of activated voxels in contralateral SMC was significantly greater in the treatment group than in the control group (*F*_group(1,75)_ = 4.071,*  P* = 0.047, Table 2). There was a significant main effect of condition for the number of activated voxels in contralateral SMC (*F*_condition  (3,75)_ = 61.694, *P* < 0.01, Table 2). Post hoc comparisons revealed that the number of activated voxels in the contralateral SMC was significantly greater for ME using affected hand than MI using affected hand (*P* < 0.01). The number of activated voxels in contralateral SMC was significantly greater for MI using affected hand than for MI using unaffected hand in the treatment group (*P* < 0.01), and the number of activated voxels was significantly greater for MI using affected hand than MI using unaffected hand in the control group (*P* < 0.05). The number of activated voxels in contralateral SMC for each condition after training is shown in Table 2.

For posttraining minus pretraining in both groups, there was a significant main effect of training for the number of activated voxels in contralateral SMC (*F*_(1,154)_ = 9.558, *P* < 0.01), and the number of activated contralateral SMC voxels of posttraining was more than that of pretraining. There was a main effect of group for the number of activated voxels in contralateral SMC (*F*_group(1,75)_ = 4.629, *P* = 0.035, Table 2), and the number of activated voxels in contralateral SMC (posttraining minus pretraining) was significantly greater in the treatment group than in the control group. However, there was no significant main effect within four conditions (*F*_condition(3,75)_ = 0.379, *P* = 0.768, Table 2).

### 3.5. Correlation Analysis for the Number of Activated Voxels in Contralateral SMC and the Change in Hand Function Score

The number of activated voxels in contralateral SMC and the change in ARAT score were positively correlated in the condition of ME using affected hand. With MI using affected hand, there was a positive correlation between the numbers of activated voxels in contralateral SMC and the change in hand function score for the MP_MI plus group. For ME and MI using unaffected hand, there was no significant correlation between these two parameters.

## 4. Discussion

The motor cortex is a region of the brain essential for the execution, learning, and control of human movement. A stroke in this area is associated with varying degrees of dyskinesia [32]. The NDT-bobath therapy is a prominent method used for rehabilitation training after stroke [33], and it may work by promoting plasticity in the central nervous system. We used NDT-bobath training in MP_MI plus PP and PP alone groups and found that after four weeks of training, hand function improved. In hemiplegic patients, treatment of the affected hand could functionally integrate the affected and unaffected hands [34].

Stroke patients, in general, do not use their affected arms, even when capable of doing so. Interestingly, MP_MI increases affected arm use [35], thus overcoming this movement suppression phenomenon [9]. Here, we showed that MP_MI training combined with PP training was better for the restoration of hand function than PP alone. In the treatment group, the increase in the number of voxels in contralateral SMC was correlated with better hand recovery. This finding was similar to a retrospective analysis study that showed that MP_MI combined with other treatments improved upper extremity function better than a single treatment [36].

During stroke rehabilitation, the areas of activated motor cortex gradually evolved. As confirmed in an earlier study, ME of a simple movement in healthy people mainly activated the contralateral SMC and the contralateral SMA [37]. During a finger-thumb opposition task, stroke patients were differed from control healthy participants in showing greater activation in the ipsilateral SMC, ipsilateral posterior parietal, and bilateral prefrontal regions. The contralateral SMC is an advanced control center for movement function and is responsible for contralateral limb movement. Our results indicated before and after training that ME using the affected and unaffected hand activated the contralateral SMC area. In patients with stroke, the activation intensity of the contralateral SMC was positively correlated with improvement in motor function [16, 32, 38]. Our results demonstrated that ME using the affected hand increased the number of voxels activated in the contralateral SMC and was associated with better motor recovery of hand function. Marshall et al. [24] demonstrated that activation in SMC from early contralesional activity to late ipsilesional activity suggested that a dynamic bihemispheric reorganization of motor networks occurred during recovery from hemiparesis.

There is some controversy surrounding MI associated functional changes and its connection to activated cortical regions. Lotze et al. were the first to demonstrate activation of the SMC with MI [39], and a large number of subsequent fMRI studies have confirmed this initial finding [22, 40]. MI of fingers, tongue, and toes activated a particular area of SMC, and only MI of fingers activated the SMC area representing fingers. Spiegler et al. [41] found that MI of tongue activated coupled neuronal networks in areas of SMC. In addition, there is evidence for hemispheric differences during MI. In right-handed subjects, MI of ipsilateral and contralateral hand movement generated left-side motor cortical activation, and MI of contralateral hand movement generated right-side motor cortical activation [42]. It was shown that MI not only activated the related cortical regions but also changed the intensity of excitation in those areas. These significant discoveries provide a strong framework for the application of MI clinically [23].

Prior to training, MI of hand movement, especially of the affected hand, mainly activated the SMA area, and after training it activated SMC. Especially under the condition of MI using the affected hand there was more activation of the contralateral SMC area. Brain activation of MI using affected hand had some differences compared to control healthy subjects. Unlike control healthy subjects and the unaffected hand, activation in primary motor cortex (BA4a) and dorsal premotor was not lower during MI as compared with ME [25]. There is some evidence that the motor functional improvement and increased attention for the affected limb in MI may be due to activation of the contralateral SMC [28]. Indeed, we found that enhanced recovery of hand function with MI was directly related to the number of activated voxels in contralateral SMC. Sharma et al. confirmed that in well-recovered subcortical stroke, the motor system including contralateral SMC was activated during MI despite the lesion, which could be targeted by rehabilitation in more severely affected patients. Wriessnegger et al. [43] also demonstrated that only 10 min of training were enough to boost MI patterns in motor related brain regions including premotor cortex and SMA and also frontoparietal and subcortical structures. These results confirmed that MI had beneficial effects especially in combination with ME when used in motor rehabilitation or motor learning processes. However, the small sample of participants in each group and lack of follow-up data were the limitation of our experiment. Future studies should focus on the changes of contralateral SMC at 3 months after MP using MI.

To enhance activation of hand motor cortex in the contralateral areas of stroke patients, we suggest implementing MP_MI training of the affected hand. MP_MI plus PP training improves hand function more than PP alone and is a promising therapeutic strategy for promoting motor recovery in stroke patients.

## Supplementary Material

The video is presented with the process of motor execution with an unaffected hand for two times in the first person perspective. The process is performed by making a fist, extending the hand and repeating one time. The process of motor imagery is similar as motor execution but have no obvious motor output.

## Figures and Tables

**Table 1:** Clinical data of all participants.

Participants	Age, years	Sex	The time since stroke, months	Handedness score	ARAT score before training
T1	39	F	2.5	10	41
T2	50	M	1.2	10	2
T3	49	F	0.8	10	1
T4	37	M	2.0	10	5
T5	47	F	2.2	10	21
T6	47	M	0.6	10	6
T7	50	M	3.0	10	52
T8	60	F	1.8	10	4
T9	52	F	2.4	10	7
T10	58	M	2.6	10	36
C1	43	F	2.5	10	25
C2	64	M	1.5	10	3
C3	64	F	2.0	10	4
C4	50	M	1.3	10	4
C5	35	M	1.9	10	6
C6	43	F	1.5	10	9
C7	66	M	2.2	10	17
C8	53	M	2.4	10	37
C9	56	M	0.5	10	1
C10	57	F	2.5	10	45

F indicates female; M, male; T, the treatment group; C, the control group; handedness score, 10 scores mean ten activities of daily performed by right hand.

**Table 2:** Hand function and the number of activated voxels in contralateral SMC under the four conditions before and after training.

Group		Pretraining		Posttraining		Posttraining-pretraining	
ARAT score	MP + PP	17.50 ± 18.83	*P* = 0.538, df = 18	30.10 ± 17.43	*P* = 0.042^*^, df = 18	12.70 ± 7.22	*P* = 0.001^**^, df = 18
	PP	15.10 ± 15.59		19.30 ± 15.12		5.20 ± 2.04	

Contralateral-SMC AM	MP + PP	825.30 ± 306.65		1224.40 ± 326.63		404.10 ± 253.50	
	PP	813.80 ± 237.77		944.60 ± 312.73		150.80 ± 230.87	
Contralateral-SMC AI	MP + PP	513.30 ± 265.77		862.80 ± 267.28		349.50 ± 304.05	
	PP	530.10 ± 149.35	*F*_ group(1,75)_ = 0.01, *P* = 0.922; *F*_condition(3,75)_ = 65.64, *P* < 0.001	684.80 ± 150.46	*F*_ group(1,75)_ = 4.07, *P* = 0.047; *F*_condition(3,75)_ = 1.69, *P* < 0.001	155.90 ± 219.82	*F*_ group(1,75)_ = 4.63, *P* = 0.035; *F*_condition(3,75)_ = 0.38, *P* = 0.768
Contralateral-SMC UM	MP + PP	1265.50 ± 298.04		1472.80 ± 313.77		206.30 ± 230.53	
	PP	1246.80 ± 322.78		1377.70 ± 286.16		130.90 ± 240.12	
Contralateral-SMC UI	MP + PP	252.00 ± 123.88		440.40 ± 135.77		188.40 ± 136.87	
	PP	289.50 ± 211.91		323.20 ± 258.10		33.70 ± 72.27	

Note: df = degree of freedom; after training, ARAT score between the MP + PP group and the PP group was compared; ^*^ *P* < 0.05, ^**^ *P* < 0.01; posttraining-pretraining = the difference between posttraining and pretraining; contralateral-SMC AM = the number of activated voxels in contralateral SMC when ME using affected hand; contralateral-SMC AI = the number of activated voxels in contralateral SMC when MI using affected hand; contralateral-SMC UM = the number of activated voxels in contralateral SMC when ME using unaffected hand; contralateral-SMC UI = the number of activated voxels in contralateral SMC when MI using unaffected hand.
